# Physical frailty and decline in general and specific cognitive abilities: the Lothian Birth Cohort 1936

**DOI:** 10.1136/jech-2019-213280

**Published:** 2019-11-05

**Authors:** Catharine Gale, Stuart J Ritchie, John M Starr, Ian J Deary

**Affiliations:** 1 Psychology, The University of Edinburgh, Edinburgh, UK; 2 MRC Lifecourse Epidemiology Unit, University of Southampton, Southampton, United Kingdom; 3 Social, Genetic and Developmental Psychiatry Centre, King's College London, London, UK; 4 Geriatric Medicine Unit, University of Edinburgh, Edinburgh, United Kingdom

**Keywords:** cohort studies, ageing, cognition

## Abstract

**Background:**

Physical frailty is associated with many adverse outcomes including disability, chronic disease, hospitalisation, institutionalisation and death. It is unclear what impact it might have on the rate of normal cognitive ageing. We investigated whether physical frailty was related to initial level of, and change in, cognitive abilities from age 70 to 79 years.

**Method:**

Participants were 950 members of the Lothian Birth Cohort 1936. Physical frailty was assessed at age 70 years using the Fried criteria. Cognitive function was assessed at ages 70, 73, 76 and 79 years. We used linear regression to examine cross-sectional and prospective associations between physical frailty status at age 70 years and factor score estimates for baseline level of and change in four cognitive domains (visuospatial ability, memory, processing speed and crystallised ability) and in general cognitive ability.

**Results:**

Physical frailty, but not prefrailty, was associated with lower baseline levels of visuospatial ability, memory, processing speed and general cognitive ability after control for age, sex, education, depressive symptoms, smoking and number of chronic illnesses. Physical frailty was associated with greater decline in each cognitive domain: age-adjusted and sex-adjusted standardised regression coefficients (95% CIs) were: −0.45 (−0.70 to –0.20) for visuospatial ability, −0.32 (−0.56 to –0.07) for memory, −0.47 (−0.72 to −0.22) for processing speed, −0.43 (−0.68 to –0.18) for crystallised ability and −0.45 (−0.70 to –0.21) for general cognitive ability. These associations were only slightly attenuated after additional control for other covariates.

**Conclusion:**

Physical frailty may be an important indicator of age-related decline across multiple cognitive domains.

## Introduction

Physical frailty is a clinical syndrome of later life characterised by weakness, slow walking speed, low activity, exhaustion and loss of weight.^1^ Its prevalence increases markedly with age: a survey of people aged 60 years or over in the English Longitudinal Study of Ageing estimated that 6.5% of those aged 60–69 years were physically frail, rising to 65% of those aged 90 years or over.^2^ People who are physically frail are at higher risk of disability, chronic disease, being admitted to hospital or a care home, and earlier death.

One potential correlate of physical frailty that has been understudied is its association with the rate of normal cognitive ageing. Most of the longitudinal evidence on the relationship between physical frailty and cognitive ageing concerns the severe end of the spectrum of cognitive decline. Thus, people who are physically frail are at higher risk of developing dementia^3^ or mild cognitive impairment^4^ (often the precursor to dementia) and are more likely to show a worsening in performance over time on screening tests for cognitive impairment such as the Mini-Mental State Examination (MMSE).^5–7^ So far as we know, only two studies have examined the relationship between physical frailty and the rate of decline of general cognitive ability or of specific cognitive domains in those who are experiencing non-pathological cognitive ageing, measured on normal-range cognitive tests. Their findings have been inconsistent: one study found that physical frailty was associated with a faster rate of decline in general cognitive ability and in episodic memory, semantic memory, working memory, perceptual speed and visuospatial abilities,^4^ but there was no control for the potential confounding influence of depressive symptoms or chronic physical diseases. Another, larger study found no associations between physical frailty and rate of change in performance on tests assessing a range of cognitive domains, although frailty was linked with poorer baseline performance on some tests.^8^

The Lothian Birth Cohort 1936 (LBC1936) was set up with the main aim of studying individual differences in non-pathological cognitive ageing.^9^ For the current study, we used four waves (at mean ages 70, 73, 76, 79 years) of cognitive data on multiple tests of processing speed, memory, visuospatial ability, crystallised cognitive ability and general cognitive ability. We tested how physical frailty or prefrailty related to baseline level and change in these domains of cognitive function and in general cognitive ability, all estimated using latent variable modelling.

## Methods

### Participants

The LBC1936 was established to study individual differences in cognitive ageing in surviving members of the Scottish Mental Survey of 1947.^9–11^ One thousand ninety-one community-dwelling people were recruited when they were on average 70 years old. Three further follow-up surveys have taken place: Wave 2 (mean age 72 years), Wave 3 (mean age 76 years) and Wave 4 (mean age 79 years). All participants gave written informed consent. The research was conducted according to the principles embodied in the Declaration of Helsinki.

### Measures

#### Physical frailty

We assessed physical frailty status as part of the Wave 1 survey when participants were on average 70 years old. This was done according to the Fried frailty phenotype.^12^ This consists of five components: slow walking speed, weakness, unintentional weight loss, self-reported exhaustion and low physical activity. People are considered to be physically frail if they have three or more of these components and prefrail if they have one or two of them.

Walking speed was indicated by how long it took participants to walk 6 m as fast as possible. We used a dynamometer to measure their maximum grip strength three times with each hand, taking the best measurement for use in analysis. Body mass index (BMI) was calculated as weight (in kilograms)/height (in metres).^2^ Participants reported how physically active they usually were on a 6-point scale, which ranged from ‘moving only in connection with necessary (household) chores’ to ‘keep-fit/heavy exercise or competitive sport several times a week’. They also completed the depression subscale of the Hospital Anxiety and Scale (HADS-D).^13^ We used definitions similar to those used by Fried to operationalise the components of frailty^12 14^: weakness was considered present if maximum grip strength was in the lowest 20% of the distribution accounting for sex and BMI; exhaustion was considered present if the participant answered ‘yes’ to the question ‘I feel as if I’m slowed down’ in the HADS-D; slow walking speed was considered present if walking speed was in the lowest 20% of the distribution accounting for sex and height; no data were available on unintentional weight loss so this was considered present if current BMI was <18.5 kg/m^2^, as in an earlier study^14^; participants whose physical activity was in the lowest sex-specific 20% of the distribution were considered to have low activity.

#### Cognitive abilities

Participants took various cognitive tests, administered in the same way at each wave. These were used as measures of four domains of ability. Tests of Matrix Reasoning and Block Design from the Wechsler Adult Intelligence Scale (WAIS-III^UK^)^15^ and Spatial Span Forwards and Backwards from the Wechsler Memory Scale (WMS-III^UK^)^16^ were used to assess visuospatial ability. Tests of Logical Memory and Verbal Paired Associates from the WMS-III^UK^ and Digit Span Backwards from the WAIS-III^UK^ were used to assess verbal-declarative memory (henceforth Memory). Tests of Digit-Symbol Substitution and Symbol Search from the WAIS-III^UK^, 4-Choice Reaction Time^17^ and Inspection Time were used to assess processing speed (henceforth Speed)^18^; of these, Inspection Time is the only test that does not need speeded responses (in their own time participants choose which of the two figures has just appeared on screen). The National Adult Reading Test (NART)^19^ and the Wechsler Test of Adult Reading (WTAR) were used to assess *c*rystallised cognitive ability.^20^ The MMSE was used to detect participants with probable cognitive impairment or dementia so we could run a sensitivity analysis to examine the effect on our results of excluding them. Except for three of the four tests of processing speed that needed fast motor responses, physical function was irrelevant for completing the tests. Descriptive statistics for each cognitive test at each wave are shown in online supplementary table 1. Online supplementary table 2 shows descriptive statistics for each cognitive test at each wave for those participants who completed all four waves.

10.1136/jech-2019-213280.supp2 Supplementary data

10.1136/jech-2019-213280.supp3 Supplementary data

#### Covariates

We chose age, years in full-time education, smoking status (categorised as never, ex-smoker and current smoker), depressive symptoms and number of chronic physical illnesses at Wave 1 as potential confounding variables. Participants completed the depression subscale of the HADS-D as a measure of depressive symptoms.^13^ As we had used data on the item ‘I feel as if I’m slowed up’ to indicate the exhaustion component of the frailty phenotype, we did not use this item when deriving score for depressive symptoms. Participants indicated whether they received a diagnosis of various chronic diseases (cancer, high blood pressure, arthritis, diabetes, cardiovascular disease or stroke). The sum of illnesses diagnosed was used as a measure of extent of morbidity.^21^

### Statistical analysis

Using structural equation modelling, the cognitive tests were divided into the latent domains of visuospatial ability, memory, speed and crystallised ability. For each domain of ability, we estimated the intercept factor (baseline level) and the slope factor (change over time). Specifically, we used a ‘factors of curves’ model,^22^ in which we estimated a latent growth curve for every individual test, then factor analysed the intercepts and slopes of these growth curves so that an overall latent ‘general’ level and an overall latent ‘general’ slope factor were produced. The idea of the latent general factor of age-related cognitive decline is well replicated across many studies, as shown in a recent meta-analysis.^23^

In a previous paper, using the first three waves of the LBC1936, we estimated a similar model^24^; here, we used the additional, fourth wave of data to extend this analysis. We estimated the four cognitive domains listed above, as well as the general factors of level and slope indicated by their intercorrelations. The factor score estimates for each of the domains and for the overall general factor were extracted from the model and used in the linear regression analyses described below. All structural equation models were produced using full information maximum likelihood estimation; these analyses were performed in Mplus V.7.3.^25^

STATA V.13 was used for the remaining analyses.^26^ All cognitive scores were standardised to facilitate comparison between different domains. We used linear regression to calculate mean differences in the overall level of each cognitive domain (the intercept at mean age 70 years) and in the slope of its change across the four measurement waves (the trajectory between ages 70 and 79 years) according to frailty status at age 70 years, using participants who were not frail as the reference group. Relationships did not vary by sex (p for interaction terms all >0.6), so we pooled the data and adjusted for sex. Regression models were adjusted for age and sex, and then additionally for the other covariates. We repeated analyses after excluding those whose MMSE score was <24, a standard cut-off that indicates possible dementia-related impairment, at any wave.^27^

## Results

Analyses were based on 950 LBC1936 participants (482 females) who had data on all the variables of interest at Wave 1 (age 70 years) baseline. Table 1 shows the characteristics of the 950 study participants according to physical frailty status at age 70 years. People who were frailer tended to be older, less educated, had more chronic disease, had a higher depressive symptom score, were more likely to smoke, had lower mean scores for each cognitive domain and in general cognitive ability at baseline and had a greater mean decline in each cognitive domain and in general cognitive ability between ages 70 and 79 years.

**Table 1:** Characteristics of the study participants according to physical frailty status at age 70 years (n=950)

Characteristic	Not frail (n=434)	Prefrail (n=444)	Frail (n=72)	P for difference
Age (years), mean (SD)	69.4 (0.87)	69.6 (0.81)	69.5 (0.76)	0.017
Female, no (%)	217 (50.0)	227 (51.1)	38 (52.8)	0.893
Years of full-time education, median (IQR)	10 (10–12)	10 (10–11)	10 (10–10)	0.0001
Smoking status, no (%)				0.008
Never	217 (50.0)	199 (44.8)	28 (38.9)	
Ex	184 (42.4)	196 (44.1)	29 (40.3)	
Current	33 (7.60)	49 (11.0)	15 (20.8)	
Depressive symptom score, median (IQR)	1 (0–2)	1 (0–3)	3 (1–5)	0.0001
Number of chronic physical illnesses, median (IQR)	1 (0–1)	1 (0–2)	2 (1–2)	0.0001
Cognitive factor score estimates for level, mean (SD)				
Visuospatial ability	0.15 (0.95)	−0.04 (1.01)	−0.67 (0.92)	<0.0001
Memory	0.13 (0.96)	−0.03 (1.02)	−0.60 (0.95)	<0.0001
Speed	0.17 (0.95)	−0.04 (0.98)	−0.77 (1.05)	<0.0001
Crystallised ability	0.12 (0.97)	−0.05 (1.00)	−0.45 (1.08)	<0.0001
General cognitive ability	0.16 (0.94)	−0.04 (1.01)	−0.70 (0.96)	<0.0001
Cognitive factor score estimates for slope, mean (SD)				
Visuospatial ability	0.07 (0.96)	−0.006 (1.03)	−0.39 (0.95)	0.002
Memory	0.05 (1.02)	−0.001 (0.99)	−0.27 (0.86)	0.041
Speed	0.07 (0.95)	−0.003 (1.04)	−0.40 (0.97)	0.001
Crystallised ability	0.08 (0.96)	−0.02 (1.04)	−0.36 (0.96)	0.003
General cognitive ability	0.07 (0.96)	−0.01 (1.03)	−0.39 (0.95)	0.001

**Figure 1:**
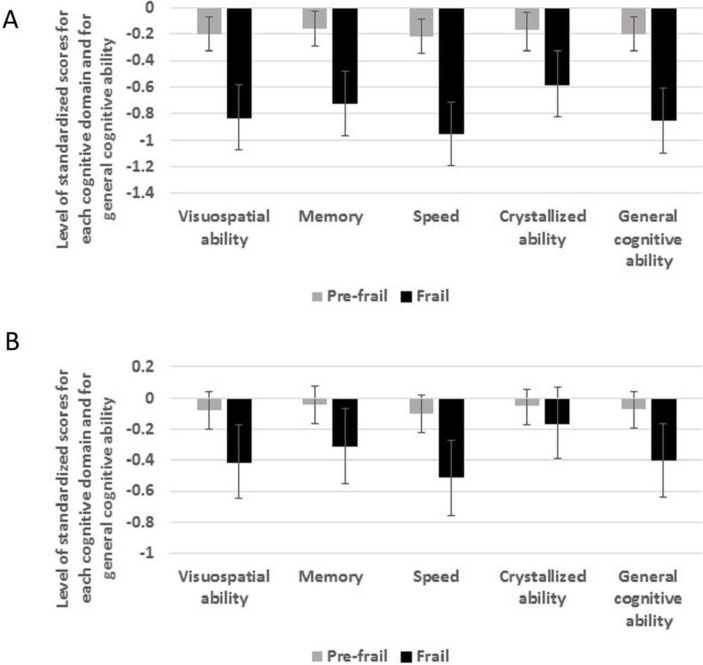
(A, B) Level of cognitive abilities at age 70 years in people who were physically frail or prefrail compared with those who were not frail. Estimates are regression coefficients (95% CIs) and have been adjusted for age and sex (A) and all covariates (B).

Table 2 and figure 1 show estimates from multivariable linear regression analyses of standardised factor score estimates of level of each cognitive domain and general cognitive ability at age 70 years according to physical frailty status. In age-adjusted and sex-adjusted analyses, people who were prefrail or frail had lower scores in each cognitive domain and in general cognitive ability. Scores in prefrail individuals were lower by between 0.17 and 0.22 of an SD; scores in frail individuals were lower by between 0.58 and 0.95 of an SD. After additional adjustment for smoking, years of education, chronic diseases and depression score at baseline, all these associations were attenuated. The associations between prefrailty and cognitive scores were attenuated by 55% (speed) to 71% (crystallised ability) and all of them ceased to be statistically significant. The associations between frailty and cognitive scores were attenuated by between 46% (speed) and 72% (crystallised ability). The latter association ceased to be significant, but people who were frail still had significantly lower scores for visuospatial ability, memory, speed and general cognitive ability than those who were not frail. Fully adjusted effect sizes ranged from 0.31 to 0.51 of an SD.

**Table 2:** Regression coefficients (95% CIs) for the relationship between physical frailty status and standardised factor score estimates of level of cognitive ability at age 70 years

Adjustments	Physical frailty status	Visuospatial ability	Memory	Speed	Crystallised ability	General cognitive ability
Age and sex	Not frail	Reference	Reference	Reference	Reference	Reference
	Prefrail	−0.20 (−0.33 to –0.07)	−0.16 (−0.29 to –0.03)	−0.22 (−0.34 to –0.09)	−0.17 (−0.31 to –0.04)	−0.20 (−0.33 to –0.07)
	Frail	−0.83 (−1.07 to –0.58)	−0.72 (−0.97 to –0.48)	−0.95 (−1.19 to –0.71)	−0.58 (−0.82 to –0.33)	−0.85 (−1.10 to –0.61)
Multivariable*	Not frail	Reference	Reference	Reference	Reference	Reference
	Prefrail	−0.08 (−0.20 to 0.04)	−0.04 (−0.16 to 0.08)	−0.10 (−0.22 to 0.02)	−0.05 (−0.17 to 0.06)	−0.07 (−0.19 to 0.04)
	Frail	−0.41 (−0.65 to –0.17)	−0.31 (−0.55 to –0.07)	−0.51 (−0.76 to –0.27)	−0.16 (−0.39 to 0.07)	−0.40 (−0.64 to –0.16)

*Age, sex, smoking status, years of education, number of chronic physical diseases and depressive symptom score.

**Figure 2:**
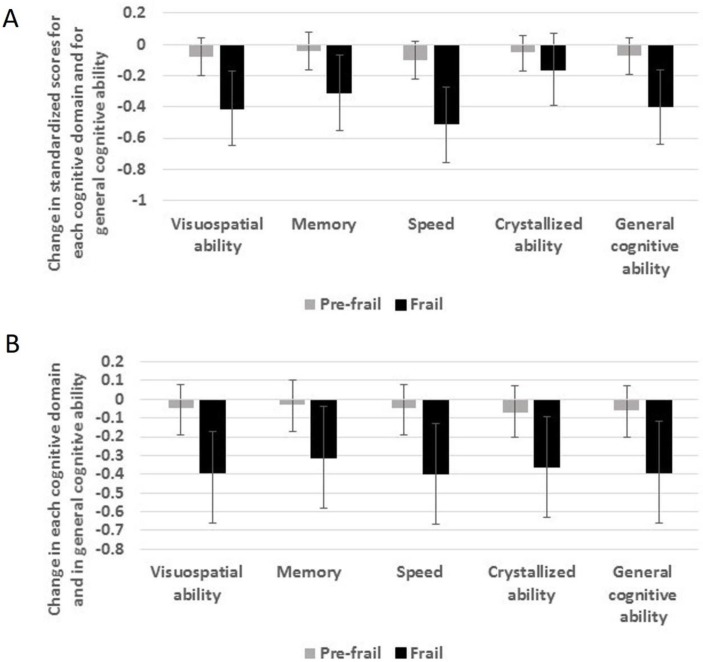
(A, B) Change in cognitive abilities between ages 70 and 79 years in people who were physically frail or prefrail at age 70 years compared with those who were not frail. Estimates are regression coefficients (95% CIs) and have been adjusted for age and sex (A) and all covariates (B).

Table 3 and figure 2 show estimates from multivariable linear regression analyses of standardised factor score estimates of change in each cognitive domain and in general cognitive ability between ages 70 and 79 years according to physical frailty status at age 70 years. After adjustment for age and sex, people who were frail, but not those who were prefrail, had greater decline in each cognitive domain and in general cognitive ability. In general, the effect sizes were highly consistent, with scores for visuospatial ability, speed, crystallised ability and general cognitive ability declining by between 0.43 and 0.47 of an SD more in frail individuals compared with those who were not frail; scores for memory declined by 0.32 of an SD more in frail individuals compared with those who were not frail. After additional adjustment for the other covariates, the associations between being physically frail and greater decline in each cognitive domain and in general cognitive ability were only slightly attenuated—by between 3% (memory) and 16% (crystallised ability)—and all remained statistically significant. Online supplementary figure 1 shows model-implied ageing trajectories for each cognitive score, grouped by frailty status.

10.1136/jech-2019-213280.supp1 Supplementary data

**Table 3:** Regression coefficients (95% CIs) for the relationship between physical frailty status and standardised factor score estimates of slope of cognitive ability between ages 70 and 79 years

Adjustments	Physical frailty status	Visuospatial ability	Memory	Speed	Crystallised ability	General cognitive ability
Age and sex	Not frail	Reference	Reference	Reference	Reference	Reference
	Prefrail	−0.07 (−0.21 to 0.06)	−0.04 (−0.17 to 0.10)	−0.07 (−0.20 to 0.06)	−0.08 (−0.22 to 0.05)	−0.08 (−0.21 to 0.06)
	Frail	−0.45 (−0.70 to –0.20)	−0.32 (−0.56 to –0.07)	−0.47 (−0.72 to –0.22)	−0.43 (−0.68 to –0.18)	−0.45 (−0.70 to –0.21)
Multivariable*	Not frail	Reference	Reference	Reference	Reference	Reference
	Prefrail	−0.05 (−0.19 to 0.08)	−0.03 (−0.17 to 0.10)	−0.05 (−0.19 to 0.08)	−0.07 (−0.20 to 0.07)	−0.06 (−0.20 to 0.07)
	Frail	−0.39 (−0.66 to –0.17)	−0.31 (−0.58 to –0.04)	−0.40 (−0.67 to –0.13)	−0.36 (−0.63 to –0.09)	−0.39 (−0.66 to –0.12)

*Age, sex, smoking status, years of education, number of chronic physical diseases and depressive symptom score.

To check whether these associations were concentrated in participants with possible dementia or cognitive impairment, we re-ran our analyses removing participants who had scored <24 on the MMSE at any wave (n=28). Results did not substantially differ from those shown in tables 2–3, indicating that potential dementia status was not a major confounding factor for the analyses described here.

## Discussion

In this 9-year longitudinal study of 70-year-0ld men and women whose cognitive function was assessed four times at 3 yearly intervals, those who were physically frail as defined by the Fried phenotype scored lower on baseline tests of visuospatial ability, memory, speed and general cognitive ability than those who were not frail by between 0.3 and 0.5 of an SD. Being physically frail was associated with a greater decline in visuospatial ability, memory, speed, crystallised ability and general cognitive ability over the follow-up period, with effect sizes ranging from 0.3 to 0.4 of an SD. These associations persisted after the exclusion of those whose scores on the MMSE were indicative of possible dementia or cognitive impairment.

Our observation that physical frailty is predictive of greater decline in various domains of cognitive function and in general cognitive ability is consistent with observations in the Rush Memory and Aging Project that physical frailty was associated with a faster decline in global cognitive function and in visuospatial ability, working memory, episodic memory, semantic memory and perceptual speed.^4^ The focus of this study was the relationship between physical frailty and incident diagnoses of mild cognitive impairment. Whether the associations found were present in those without mild cognitive impairment or whether they survived control for the potential confounding effect of disease burden or depression is unclear.^4^ Here, we found that the associations between physical frailty and greater decline in each cognitive domain examined and in general cognitive ability were little changed by the exclusion of those whose scores on the MMSE suggested possible dementia or cognitive impairment, and the associations were independent of number of chronic diseases present, depressive symptoms and other covariates. In the only other study to examine whether physical frailty was linked with decline in specific cognitive domains, no significant associations were found.^8^ Based on the Canberra Longitudinal Study, it had four waves of data spanning a 12-year follow-up. One potential limitation was that, in contrast to the current study, each cognitive domain was assessed using the scores from a single test rather than with general factors derived from two or more tests so may provide a less accurate, or more error-prone, indication of participants’ abilities in that domain.

How could future research increase our understanding of the link between physical frailty and cognitive decline? One priority should be to examine changes in physical frailty and changes in cognitive abilities together in longitudinal panels to investigate lead-lag effects, that is, whether changes in cognitive abilities follow changes in physical frailty, or vice versa, or whether there are reciprocal effects. Evidence from a study of two US cohorts over an average period of 6 years showed that there is a strong correlation between rates of change in physical frailty and in cognitive ability (ρ=−0.73).^28^ The findings of the current study, coupled with those of a previous study in the LBC1936 cohort which showed that lower levels of and greater decline in the major domains of cognitive ability were associated with greater risk of the onset of physical frailty,^29^ suggest that the relationship between physical frailty and cognitive ability in later life may be bi-directional. Another area for exploration is whether genetic susceptibility to physical frailty is associated with differences in cognitive ageing. Twin studies have reported heritability estimates for physical frailty of around 40%,^30 31^ however, in the small cohorts studied to date, no genetic variant has been consistently associated with this phenotype. Meta-analysis of genome-wide association studies of physical frailty should throw light on which genetic variants confer susceptibility, allowing the construction of polygenic risk scores. In the current study, chronic physical diseases, depressive symptoms, smoking and education had only a small attenuating effect on the associations between physical frailty and decline in cognitive abilities. Future studies could investigate other potential explanatory factors, such as sedentary behaviour and lack of physical activity. Scoring higher on a latent trait of physical fitness (based on lung function, grip strength and walking speed) was associated with less decline in cognitive ability in a previous study of this cohort,^24^ suggesting that being less sedentary and more physically active may have benefits for cognitive ageing. Physical activity is thought to be the most effective strategy to prevent or reduce the severity of physical frailty,^32 33^ and longitudinal studies show that people who spend less time sitting are less likely to become physically frail, regardless of their level of physical activity.^34^ Another area for further research using longitudinal data is the part played by brain neuropathology in the associations between physical frailty and cognitive ability.^28^

The main strength of our study includes the use of multiple tests to assess each domain of cognitive ability at repeated waves, which allowed us to investigate how physical frailty or prefrailty related to initial level and change in each domain and in general cognitive ability. Another strength is the narrow age range of our sample, which largely eliminates the confounding effect of age differences between participants. A weakness is that some individuals will have experienced decline in cognitive abilities and onset of physical frailty before age 70 years, so we cannot determine whether frailty precedes cognitive decline or whether both physical and cognitive abilities are declining simultaneously.

The clinical syndrome of physical frailty may be an important predictor of age-related decline across multiple cognitive domains. Future research needs to elucidate the mechanisms, whereby being physically robust seems to be protective against cognitive decline.

**What is already known on this subject**

- Older people who are physically frail have an increased risk of developing dementia or mild cognitive impairment.
- It is unclear whether physical frailty predicts the rate of decline of general cognitive ability or specific cognitive domains in people who are experiencing normal cognitive ageing.

**What this study adds**

- Older people who are physically frail have a faster rate of decline in memory, processing speed, visuospatial ability, crystallised ability and general cognitive ability.
- Smoking status, education, number of chronic diseases and depressive symptoms explained little of these associations.
- Future research should investigate the role of sedentary behaviour and lack of physical activity.
