# Supplementary material for: Physical frailty and decline in general and specific cognitive abilities: the Lothian Birth Cohort 1936
Source: J Epidemiol Community Health. 2019 Nov 5;74(2):108–13. doi: 10.1136/jech-2019-213280 (PMC6993023; doi:10.1136/jech-2019-213280)
Supplement: Supplementary data [file jech-2019-213280supp002.pdf]

Supplementary Table 1: Descriptive statistics for each cognitive test at each wave of the Lothian Birth Cohort 1936 study

Test	Wave 1 (70 years)		Wave 2 (73 years)		Wave 3 (76 years)		Wave 4 (79 years)	
	Mean (SD)	N	Mean (SD)	N	Mean (SD)	N	Mean (SD)	N
Matrix reasoning	13.49 (5.13)	1086	13.17 (4.96)	863	13.04 (4.91)	689	12.90 (5.03)	535
Block design	33.79 (10.32)	1085	33.64 (10.08)	864	32.18 (9.95)	691	31.20 (9.63)	535
Spatial span	7.36 (1.42)	1084	7.35 (1.38)	861	7.31 (1.36)	690	7.07 (1.36)	536
Logical memory	71.46 (17.96)	1087	74.30 (17.88)	864	74.58 (19.20)	688	72.71 (20.39)	542
Verbal paired associates	26.44 (9.13)	1050	27.18 (9.46)	843	26.41 (9.56)	663	27.14 (9.55)	497
Digit span backwards	7.73 (2.26)	1090	7.81 (2.29)	866	7.77 (2.37)	695	7.56 (2.18)	548
NART	34.48 (8.15)	1089	34.38 (8.18)	864	35.02 (8.03)	695	35.59 (8.19)	546
WTAR	41.02 (7.17)	1089	41.01 (6.97)	864	41.09 (7.02)	694	41.63 (7.03)	546
Verbal	42.42 (12.54)	1087	43.18 (12.94)	865	42.90 (12.76)	696	43.61 (13.33)	547
Digit-symbol substitution	56.60 (12.93)	1086	56.40 (12.31)	862	53.81 (12.93)	685	49.70 (38.06)	535
Symbol search	24.71 (6.39)	1086	24.61 (6.18)	862	24.60 (6.46)	687	19.25 (56.65)	529
Inspection time	112.14 (11.00)	1041	111.22 (11.79)	838	110.14 (12.55)	654	105.06 (43.14)	465
Choice reaction time (sec.)	642.11 (85.78)	1084	649.27 (89.84)	865	678.84 (102.75)	685	706.03 (113.61)	543

Note: Ages are rounded to the nearest year
