# Supplementary material for: Physical frailty and decline in general and specific cognitive abilities: the Lothian Birth Cohort 1936
Source: J Epidemiol Community Health. 2019 Nov 5;74(2):108–13. doi: 10.1136/jech-2019-213280 (PMC6993023; doi:10.1136/jech-2019-213280)
Supplement: Supplementary data [file jech-2019-213280supp003.pdf]

Supplementary table 2: Descriptive cognitive data for participants who completed all four waves of the study (maximal *N* = 539)

Test	Wave 1 (70 years)		Wave 2 (73 years)		Wave 3 (76 years)		Wave 4 (79 years)	
	Mean (SD)	N	Mean (SD)	N	Mean (SD)	N	Mean (SD)	N
Matrix reasoning	14.46 (5.02)	538	13.91 (4.89)	537	13.33 (4.91)	532	12.97 (5.02)	524
Block design	35.67 (10.13)	537	34.70 (10.13)	538	32.72 (9.79)	534	31.32 (9.66)	524
Spatial span	7.54 (1.37)	536	7.46 (1.34)	535	7.41 (1.34)	533	7.08 (1.37)	525
Logical memory	74.43 (16.87)	539	76.30 (16.93)	539	75.78 (18.45)	535	73.02 (20.30)	531
Verbal paired associates	27.88 (8.53)	526	28.45 (9.04)	527	27.29 (9.25)	515	27.31 (9.44)	487
Digit span backwards	8.03 (2.37)	539	8.01 (2.32)	539	7.91 (2.41)	537	7.58 (2.19)	537
NART	35.60 (7.97)	538	35.23 (7.99)	537	35.60 (7.95)	537	35.70 (8.10)	535
WTAR	41.90 (6.95)	538	41.76 (6.61)	537	41.56 (6.93)	537	41.72 (6.97)	535
Verbal fluency	43.60 (12.77)	538	44.37 (12.97)	538	43.75 (12.72)	538	43.72 (13.32)	536
Digit-symbol substitution	59.00 (12.40)	536	58.37 (11.98)	536	55.43 (12.27)	527	51.38 (13.00)	524
Symbol search	25.64 (6.58)	538	25.49 (5.92)	536	25.26 (6.26)	531	22.71 (6.73)	518
Inspection time	113.43 (10.52)	524	112.26 (11.65)	529	110.97 (11.83)	516	107.05 (13.60)	458
Choice reaction time (ms)	626.72 (79.20)	538	636.94 (82.66)	538	668.61 (94.60)	528	704.79 (113.95)	532
