# Supplementary material for: Physical frailty and decline in general and specific cognitive abilities: the Lothian Birth Cohort 1936
Source: J Epidemiol Community Health. 2019 Nov 5;74(2):108–13. doi: 10.1136/jech-2019-213280 (PMC6993023; doi:10.1136/jech-2019-213280)

Supplementary Figure 1: Model-implied ageing trajectories for each cognitive score, grouped by frailty status.

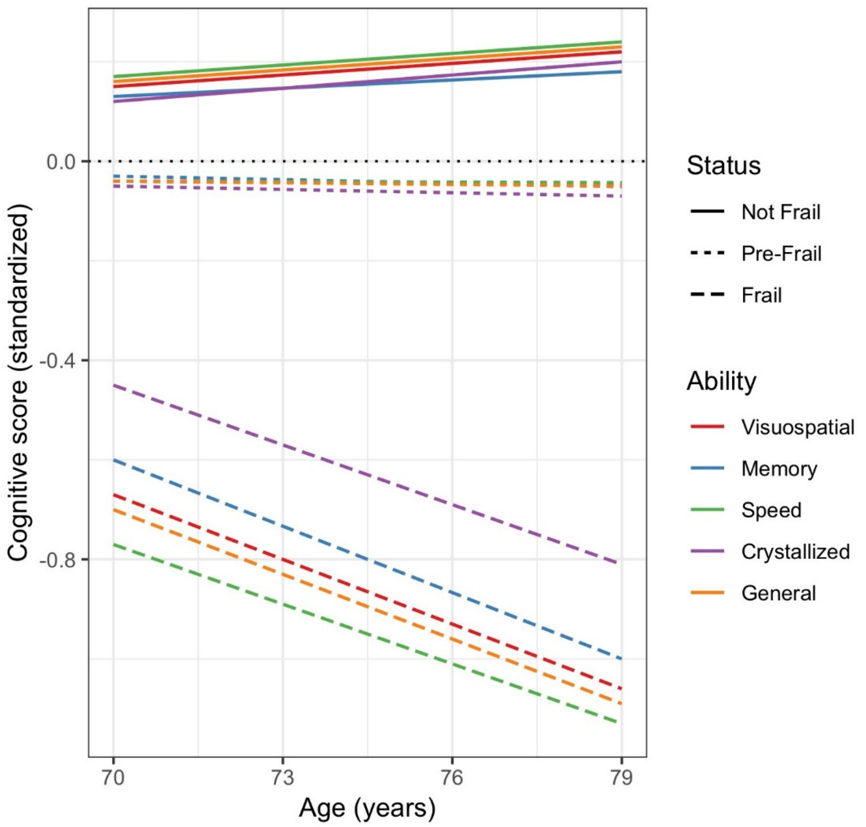

Supplement: Supplementary data [file jech-2019-213280supp001.pdf]
